# Dietary inflammation and socioeconomic status mediate depression–constipation link: a cross-sectional analysis of NHANES 2005–2010

**DOI:** 10.3389/fnut.2025.1668654

**Published:** 2025-10-01

**Authors:** Qiulu Huang, Haifang Zhou, Mei Yang, Yilin Meng, Lina Wang

**Affiliations:** ^1^Department of Nursing, Hangzhou TCM Hospital Affiliated to Zhejiang Chinese Medical University, Xihu District, Hangzhou, Zhejiang Province, China; ^2^Department of Clinical Psychology, Hangzhou TCM Hospital Affiliated to Zhejiang Chinese Medical University, Xihu District, Hangzhou, Zhejiang Province, China; ^3^Department of Nursing School, Anhui University of Chinese Medicine, Hefei, China

**Keywords:** depression, constipation, NHANES, mediation analysis, DII, PIR

## Abstract

**Background:**

This study investigated the relationship between depression and constipation and examined potential mediating roles of dietary inflammatory index (DII) and socioeconomic status using data from NHANES 2005–2010.

**Methods:**

We analyzed 12,854 adults with complete data on depression (PHQ-9), constipation (self-report/Bristol Stool Scale), DII (27 nutrients), and poverty-to-income ratio (PIR). Statistical analyses included multivariable logistic regression with appropriate reference categories, restricted cubic splines (RCS), mediation analysis, and subgroup assessments.

**Results:**

Constipated individuals exhibited significantly higher depression severity (mean PHQ-9: 4.25 vs. 3.00), higher DII (2.00 vs. 1.37), and lower PIR (all *p* < 0.0001). After adjustments, PHQ-9 scores were independently associated with constipation risk (OR: 1.04, 95% CI: 1.03–1.06), with a non-linear relationship showing an inflection point at PHQ-9 = 10 (scores <10: OR = 1.08; scores ≥10: OR = 0.98). Statistical mediation analysis revealed that DII mediated 6.03% and PIR mediated 12.46% of the depression–constipation association. Subgroup analyses demonstrated consistent associations across all demographic and clinical subgroups (OR range: 1.04–1.14).

**Conclusion:**

This cross-sectional study demonstrates a significant non-linear relationship between depression and constipation, partially mediated by dietary inflammation and socioeconomic status. Longitudinal studies are needed to establish causality and directionality between these variables.

## 1 Introduction

Gastrointestinal disorders impose substantial burdens on global healthcare systems, with chronic constipation representing a prevalent yet mechanistically complex condition that affects approximately 10–16% of adults worldwide (64). Its bidirectional relationship with depression is increasingly recognized as a critical public health concern (1). While cross-sectional studies consistently show associations between depression and functional gastrointestinal disturbances (2, 3), longitudinal research provides stronger evidence for causality. For instance, prospective cohort studies have shown that depression increases the risk of incident constipation [e.g., Liu et al. (4): HR = 1.72, 95% CI: 1.42–2.08] (2, 3). Conversely, constipation has also been shown to predict subsequent depression [Koloski et al. (5): OR = 2.36, 95% CI: 1.58–3.51]. Thus, neuroendocrine dysregulation and autonomic dysfunction may play a role in the relationship between depression and constipation (6, 7), while chronic distress from constipation may be linked to depression through reduced quality of life and impaired social functioning (8). The current evidence suggests a reinforcing cycle, though intervention studies indicate that depression treatment more often improves constipation than vice versa (3, 9).

Despite established correlations, mechanistic insights into the depression–constipation nexus remain fragmented. Existing research primarily implicates factors such as reduced colonic motility under stress-axis activation and disruptions in serotonergic pathways, such as pivotal elements linking these conditions (10, 11). Moreover, evidence suggests that alterations in gut microbiota and systemic inflammation may play critical roles in mediating this relationship, although further exploration is necessary to clarify their impact (7, 12). However, critical gaps persist in understanding intermediary mechanisms associated with both psychological distress and gastrointestinal pathology. The roles of lifestyle-modifiable mediators—specifically diet-driven systemic inflammation and socioeconomic disparities—are inadequately characterized within this relationship (13), limiting the development of targeted interventions aimed at addressing both conditions simultaneously.

Recent research highlights a compelling association between inflammation, depression, and gastrointestinal dysfunction, with particular focus on the role of pro-inflammatory cytokines associated with changes in gut neuromuscular coordination and visceral sensitivity (7, 10). This relationship is underscored by findings indicating that diet-derived inflammation, as operationalized by the DII, may be statistically associated with these relationships. Existing literature shows a positive correlation between higher DII scores and the incidence of depression, alongside gastrointestinal symptoms such as constipation (14). Despite these insights, studies specifically quantifying DII’s potential statistical mediation in the depression–constipation context remain scarce.

Moreover, the influence of socioeconomic status (SES) on depression and gastrointestinal health is significant, yet often underexplored (15, 16). Low SES has been correlated with heightened risks of depressive symptoms and comorbid conditions such as constipation, as individuals facing economic hardships frequently have limited access to quality healthcare and nutritious food (17, 18). Recent studies indicate that SES might be associated with dietary factors, both independently and interactively, in relation to the risk of constipation (19). However, comprehensive models integrating psychological, dietary–inflammatory, and socioeconomic mediators in large-scale human studies are largely lacking, which calls for further research to fill these critical gaps.

While it is generally observed that depression severity may exhibit linear relationships with somatic outcomes, the potential for non-linear dynamics—such as threshold effects in the association with constipation—is an area ripe for exploration using flexible epidemiological tools, including restricted cubic splines (20). In light of these factors, addressing these complex interrelations is crucial for developing targeted interventions for high-risk populations plagued by depression and gastrointestinal complications.

Leveraging NHANES data (2005–2010) from 12,854 U.S. adults, this study addresses these critical gaps by: (1) quantifying independent associations between depression severity, DII, and PIR with constipation risk; (2) identifying potential threshold effects using non-linear modeling; and (3) empirically decomposing the statistical mediation of diet-induced inflammation and socioeconomic disparities. Our findings suggest associations between depression, pro-inflammatory diets, and increased constipation risk, while also identifying a potential protective association with higher SES. Building upon established associations between individual factors and constipation risk, we provide the first formal statistical mediation analysis examining the relationships between depression, dietary inflammation, and socioeconomic status within a unified analytical framework using multi-mediator structural equation modeling.

## 2 Methods

### 2.1 Study population

**Figure 1:**
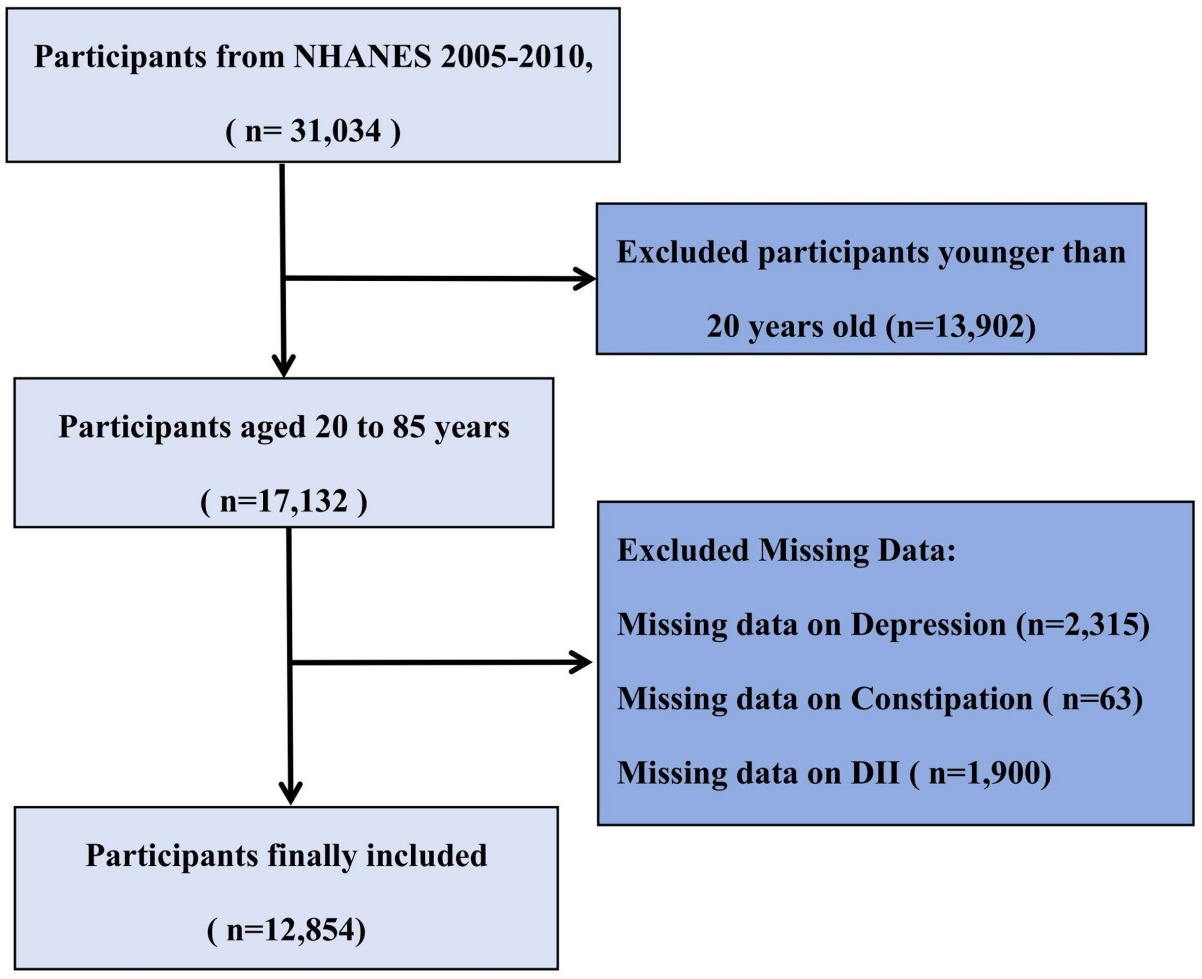
Flowchart of participant selection from NHANES 2005–2010.

The present study utilized data from the National Health and Nutrition Examination Survey (NHANES) 2005–2010 cycles because these three 2-year cycles included relevant questionnaire data on bowel health. NHANES is a comprehensive nationwide survey conducted by the National Center for Health Statistics (NCHS) to assess the health and nutritional status of the U.S. population (65, 66). It employs a complex, multistage probability sampling design to represent the non-institutionalized U.S. civilian population, with strategic oversampling of specific subgroups (elderly, African Americans, and Hispanics) to enhance estimate precision (21). Our study’s participant selection workflow is depicted in Figure 1. All procedures were approved by the NCHS Ethics Review Board, and all participants provided written informed consent. Detailed methodological information is available on the NHANES website (22). From an initial sample of 31,034 participants, we excluded 13,902 individuals younger than 20 years of age, as they did not complete the bowel health questionnaire. Among the remaining 17,132 adult participants (aged 20–85 years), we further excluded those with missing data on depression (*n* = 2,315), constipation (*n* = 63), and DII (*n* = 1,900). The final analytical cohort comprised 12,854 adults with complete data on all key variables of interest.

### 2.2 Assessment of depression

Depression was assessed using the PHQ-9 (23, 24). This nine-item questionnaire employs a 4-point rating scale (0 = not at all; 1 = several days; 2 = more than half the days; and 3 = nearly every day), with total scores ranging from 0 to 27 (25). The PHQ-9 demonstrates high sensitivity (86%) and specificity (85%) in depression screening, with a clinical cutoff score of 1,020. According to validated severity classifications 21, total scores of 0–4 indicate no depression, 5–9 indicate mild depression, 10–14 indicate moderate depression, and ≥15 indicate moderately severe to severe depression (26).

### 2.3 Assessment of constipation

Constipation assessment was based on self-reported questionnaires and the Bristol Stool Form Scale (BSFS). Following previous studies, constipation was diagnosed based on two criteria: (1) self-reported bowel movements less than three times per week and (2) stool consistency corresponding to BSFS types 1 or 2. The BSFS classifies stools into seven types, with types 1–2 categorized as constipation, types 3–5 as normal, and types 6–7 as diarrhea (27, 28). In the NHANES database, constipation was assessed by asking, “Have you experienced constipation in the past 12 months?” For analytical purposes, frequency descriptors were operationalized as follows: “often” was defined as symptoms occurring ≥3 days/week but <6 days/week, and “always” was defined as symptoms occurring ≥6 days/week, consistent with functional gastrointestinal disorder diagnostic criteria from Rome IV (29). Respondents who answered “always” or “often” were classified as having constipation (28, 30). This combined diagnostic approach (BSFS and self-report) has demonstrated good specificity (85%) with moderate sensitivity (72%) in previous validation studies when compared to clinical evaluations (31).

### 2.4 Assessment of DII

The DII was calculated based on the average dietary data obtained from two 24-h dietary recall interviews in NHANES. This index, developed by Shivappa et al. (32), assesses the inflammatory potential of diet through nutrient composition analysis, with detailed information available elsewhere (15). While the complete DII incorporates 45 nutrients, our study utilized 27 available parameters due to data availability constraints in the NHANES database, a common limitation acknowledged in studies using secondary dietary datasets (33, 34). The 27 parameters included carbohydrates; energy; protein; fat; fiber; cholesterol; saturated fatty acids; monounsaturated fatty acids; polyunsaturated fatty acids; *β*-carotene; vitamins A, B1, B2, B6, B12, C, D, and E; folate; iron; magnesium (Mg); zinc; selenium; omega-3 and omega-6 polyunsaturated fatty acids; alcohol; and caffeine. Importantly, this reduced version of the DII has been validated in prior research. For instance, a study by Wirth et al. (35) demonstrated that a DII based on 28 components significantly correlated with inflammatory biomarkers (CRP and IL-6) in a similar magnitude as the full DII. Similarly, Tabung et al. (36) reported consistent associations between a reduced DII and health outcomes. Therefore, the 27-component DII used in our study is considered a valid measure of dietary inflammatory potential. Scores ≥0 indicate pro-inflammatory diets, while scores <0 represent anti-inflammatory dietary patterns (37).

### 2.5 Assessment of PIR

PIR reflects household income relative to poverty thresholds, accounting for household size, composition, member ages, and annual inflation adjustments. These values, derived from the Department of Health and Human Services guidelines, provide precise poverty status assessments across years and states (38). For analytical purposes, income level was categorized into tertiles: low (PIR < 1.3), middle (PIR 1.3–3.5), and high (PIR ≥ 3.5) (39, 40). These cutoff points were determined based on both policy-relevant thresholds and data distribution: the lower cutoff (1.3) approximates the Supplemental Nutrition Assistance Program (SNAP) eligibility threshold (130% of the poverty line) in many states (41), while the upper cutoff (3.5) is supported by research indicating that it represents a significant threshold in health disparities analysis (42) and aligns with prevalent income classifications used in health studies (42). This categorization is consistent with several NHANES-based studies examining health disparities across socioeconomic groups (42–44).

### 2.6 Covariables

NHANES-trained interviewers used a well-designed questionnaire to collect demographic, health, and biomarker data. Covariates were selected based on previous research findings and clinical expertise (45). The covariates we gathered included age, sex, ethnicity, marital status, education level, body mass index (BMI), smoking status, alcohol consumption status, hypertension, and diabetes. Ethnicity was categorized as Mexican American, non-Hispanic White, non-Hispanic Black, other Hispanic, and other ethnicities. Marital status was classified as married/living with partner, widowed/divorced/separated, and never married. Education level was divided into less than high school, high school, and above high school. BMI was categorized as <25.0, 25.0–29.9, and ≥30.0 kg/m^2^. Smoking status was assessed using two questions from the NHANES questionnaire, with participants classified as never smokers, former smokers, and current smokers. Alcohol consumption status was categorized based on the average number of drinks per drinking day in the past 12 months as never drinkers, former drinkers, light drinkers, moderate drinkers, and heavy drinkers. Hypertension was defined as follows: (1) self-reported physician diagnosis; (2) mean diastolic blood pressure (DBP) ≥ 90 mmHg; (3) mean systolic blood pressure (SBP) ≥ 140 mmHg; or (4) current use of antihypertensive medication. Diabetes was defined as follows: (1) self-reported physician diagnosis; (2) laboratory indicators: glycated hemoglobin (HbA1c) ≥ 6.5%, fasting plasma glucose ≥7.0 mmol/L, random plasma glucose ≥11.1 mmol/L, or 2-h plasma glucose in oral glucose tolerance test (OGTT) ≥ 11.1 mmol/L; or (3) current use of diabetes medication or insulin therapy. Hyperlipidemia was determined based on self-reported medical history. Laboratory data, including total cholesterol and parameters for Fatty Liver Index (FLI) calculation, were extracted from the NHANES dataset. Total cholesterol and high-density lipoprotein cholesterol (HDL-C) were measured from fasting venous blood (≥9 h) according to NHANES protocols: enzymatically for cholesterol and via immunoinhibition/precipitation for HDL-C. The results were converted to mmol/L using a 0.02586 factor with strict quality control. Data, including these and FLI parameters, were extracted from the NHANES.

### 2.7 Statistical analysis

Between-group baseline differences were assessed using independent *t*-tests for continuous variables and chi-square tests for categorical variables. For the comparison of baseline characteristics between groups with and without constipation, we used the standard significance level (*α* = 0.05) without correction for multiple comparisons, as these analyses were exploratory in nature and intended to describe the sample characteristics rather than test formal hypotheses. Continuous variables were presented as mean ± standard deviation, and categorical variables were presented as frequencies and percentages. Missing data for key variables (<5%) were handled using multiple imputation. The associations between depression and constipation risk were evaluated using multivariable logistic regression models with three progressive models: Model 1 was unadjusted; Model 2 was adjusted for age, sex, ethnicity, and education level; and Model 3 was further adjusted for marital status, PIR, BMI, smoking status, alcohol consumption, physical activity, DII, diabetes, hyperlipidemia, hypertension, total cholesterol, HDL-C, fiber, polyunsaturated fatty acids (PUFA), vitamin D, and magnesium (Mg). The associations between DII and constipation risk and between P and constipation risk were analyzed using the same models.

To explore potential non-linear dose-response relationships between depression and constipation risk, restricted cubic spline models were applied with smooth curve fitting. A threshold effect analysis was subsequently conducted to identify the inflection point (PHQ-9 score = 10) and evaluate the presence of non-linearity, with a two-piecewise linear regression model constructed at the identified point and model fitness compared via log-likelihood ratio tests. Stratified analyses were conducted according to sex, age group (20–39, 40–59, and ≥60 years), ethnicity, education level, marital status, BMI categories (<25, 25–30, and ≥30 kg/m^2^), and PIR levels (<1.3, 1.3–3.5, and >3.5) to evaluate whether these factors modified the association between depression and constipation risk.

For the mediation analysis assessing the intermediary roles of DII and PIR in the depression–constipation relationship, we used R version 4.2.2 and implemented a counterfactual framework-based causal mediation analysis via the ‘mediation’ package. The existence of a significant mediation effect was defined by satisfying all of the following criteria: (1) a statistically significant indirect effect (average causal mediation effect, ACME; *p* < 0.05), (2) a statistically significant total effect (*p* < 0.05), and (3) a positive proportion mediated effect (ACME/total effect). To account for the complex survey design of the NHANES (including clustering, stratification, and sampling weights), we performed the statistical mediation analysis within the survey framework using the ‘survey’ package. Specifically, we created a survey design object incorporating sampling weights, primary sampling units, and strata. Bootstrap resampling (1,000 replicates) was applied to estimate 95% confidence intervals for mediation effects, ensuring consistent handling of the complex design features. All mediation analyses were adjusted for the same covariates included in Model 3 of the logistic regression analyses.

All analyses were conducted using the NHANES complex multistage sampling framework. Analyses were performed using R software (version 4.2.2) for primary analyses and mediation modeling, EmpowerStats (version 2.0) with the rms package for restricted cubic spline models, and Stata for supplementary analyses. Statistical significance was defined as a *p*-value of <0.05 (two-sided).

## 3 Results

### 3.1 Baseline characteristics

Table 1 presents the baseline characteristics of 12,854 participants stratified by constipation status. Participants with constipation (n = 1,334, 10.4% of total) were significantly younger (46.95 ± 18.59 vs. 50.28 ± 17.84 years, *p* < 0.001) and predominantly women (73.54% vs. 49.44%, *p* < 0.001). The constipated group included a higher proportion of non-Hispanic Black (25.86% vs. 18.68%, *p* < 0.001) participants and showed lower educational attainment (41.10% vs. 49.93% above high school, *p* < 0.001), were less likely to be married (56.97% vs. 62.96%, *p* < 0.001), and had a greater proportion in the lowest family poverty-to-income ratio category (36.81% vs. 27.67%, *p* < 0.001). Regarding lifestyle and clinical factors, participants with constipation were more likely to be never-smokers (58.10% vs. 52.18%, *p* < 0.001) and never-drinkers (18.44% vs. 12.91%, *p* < 0.001) and had a lower prevalence of hypertension (37.11% vs. 42.47%, p < 0.001), diabetes (16.63% vs. 18.32%, p < 0.001), and hyperlipidemia (69.34% vs. 72.74%, *p* = 0.008). Nutritional assessments revealed that the constipation group had significantly higher dietary inflammatory index scores (2.00 ± 1.60 vs. 1.37 ± 1.81, *p* < 0.001), fiber intake (0.32 ± 0.41 vs. 0.19 ± 0.46, *p* < 0.001), PUFA (0.02 ± 0.25 vs. −0.04 ± 0.25, *p* < 0.001), vitamin D (0.23 ± 0.27 vs. 0.19 ± 0.30, *p* < 0.001), magnesium (0.15 ± 0.22 vs. 0.06 ± 0.25, *p* < 0.001), and HDL-C (1.42 ± 0.43 vs. 1.37 ± 0.42 mmol/L, *p* < 0.001). Consistently, participants with constipation exhibited greater depression severity (mean PHQ-9 score: 4.25 ± 4.88 vs. 3.00 ± 4.10, *p* < 0.001) and a higher percentage of severe depression (5.55% vs. 2.89%, *p* < 0.001).

**Table 1:** Baseline characteristics of participants with or without constipation.

Characteristics	Total	Constipation		
	*n* = 12,854	No	Yes	*p*-value
		*n* = 11,520	*n* = 1,334	
Age (years)	49.93 ± 17.95	50.28 ± 17.84	46.95 ± 18.59	<0.001
Sex, *n* (%)				<0.001
Male	6,178 (48.06)	5,825 (50.56)	353 (26.46)	
Female	6,676 (51.94)	5,695 (49.44)	981 (73.54)	
Ethnicity, *n* (%)				<0.001
Non-Hispanic White	6,543 (50.90)	5,947 (51.62)	596 (44.68)	
Non-Hispanic Black	2,497 (19.43)	2,152 (18.68)	345 (25.86)	
Mexican American	2,273 (17.68)	2054 (17.83)	219 (16.42)	
Other Hispanic	1,058 (8.23)	930 (8.07)	128 (9.60)	
Other ethnicities	483 (3.76)	437 (3.79)	46 (3.45)	
Education level, *n* (%)				<0.001
Below high school	1,447 (11.27)	1,277 (11.09)	170 (12.77)	
High school	5,100 (39.71)	4,486 (38.97)	614 (46.13)	
Above high school	6,295 (49.02)	5,748 (49.93)	547 (41.10)	
Marital status, *n* (%)				<0.001
Married	8,009 (62.34)	7,249 (62.96)	760 (56.97)	
Divorced	2,842 (22.12)	2,519 (21.88)	323 (24.21)	
Never married	1996 (15.54)	1745 (15.16)	251 (18.82)	
BMI (kg/m^2^), *n* (%)				<0.001
<25	3,574 (28.05)	3,117 (27.29)	457 (34.65)	
≥25, <30	4,392 (34.47)	3,970 (34.76)	422 (31.99)	
≥30	4,775 (37.48)	4,335 (37.95)	440 (33.36)	
Family PIR, *n* (%)				<0.001
<1.3	3,418 (28.61)	2,966 (27.67)	452 (36.81)	
≥1.3, <3.5	4,618 (38.66)	4,134 (38.57)	484 (39.41)	
≥3.5	3,910 (32.73)	3,618 (33.76)	292 (23.78)	
Smoking status, *n* (%)				<0.001
Never	6,785 (52.79)	6,010 (52.18)	775 (58.10)	
Former	3,369 (26.21)	3,094 (26.86)	275 (20.61)	
Now	2,698 (20.99)	2,414 (20.96)	284 (21.29)	
Drinking status, *n* (%)				<0.001
Never	1731 (13.48)	1,485 (12.91)	246 (18.44)	
Former	2,637 (20.54)	2,340 (20.34)	297 (22.26)	
Mild	4,054 (31.57)	3,696 (32.12)	358 (26.84)	
Moderate	1883 (14.67)	1702 (14.79)	181 (13.57)	
Heavy	2,535 (19.74)	2,283 (19.84)	252 (18.89)	
Hypertension, *n* (%)				<0.001
No	7,465 (58.09)	6,626 (57.53)	839 (62.89)	
Yes	5,386 (41.91)	4,891 (42.47)	495 (37.11)	
Diabetes, *n* (%)				<0.001
No	9,134 (73.13)	8,157 (72.62)	977 (77.72)	
Diabetes mellitus	2,267 (18.15)	2058 (18.32)	209 (16.63)	
Impaired fasting glycemia	501 (4.01)	472 (4.20)	29 (2.31)	
Impaired glucose tolerance	588 (4.71)	546 (4.86)	42 (3.34)	
Hyperlipidemia, *n* (%)				0.008
No	3,549 (27.61)	3,140 (27.26)	409 (30.66)	
Yes	9,305 (72.39)	8,380 (72.74)	925 (69.34)	
Physical activity	926.58 ± 1485.81	932.02 ± 1499.28	874.59 ± 1349.86	0.277
Metabolic equivalent of task	3631.68 ± 5904.72	3651.18 ± 5957.17	3444.17 ± 5373.82	0.321
DII	1.44 ± 1.80	1.37 ± 1.81	2.00 ± 1.60	<0.001
Fiber	0.20 ± 0.46	0.19 ± 0.46	0.32 ± 0.41	<0.001
PUFA	−0.04 ± 0.26	-0.04 ± 0.25	0.02 ± 0.25	<0.001
Vitamin D	0.19 ± 0.29	0.19 ± 0.30	0.23 ± 0.27	<0.001
Mg	0.07 ± 0.25	0.06 ± 0.25	0.15 ± 0.22	<0.001
Total cholesterol (mmol/L)	5.11 ± 1.09	5.11 ± 1.09	5.12 ± 1.10	0.8520
HDL-C (mmol/L)	1.37 ± 0.42	1.37 ± 0.42	1.42 ± 0.43	<0.001
Depression score	3.13 ± 4.20	3.00 ± 4.10	4.25 ± 4.88	<0.001
Severity of depression, *n* (%)				<0.001
No depression	9,787 (76.14)	8,906 (77.31)	881 (66.04)	
Mild depression	1969 (15.32)	1713 (14.87)	256 (19.19)	
Moderate depression	691 (5.38)	568 (4.93)	123 (9.22)	
Severe depression	407 (3.17)	333 (2.89)	74 (5.55)	

Data are presented as mean ± standard deviation for continuous variables and number (percentage) for categorical variables. *p*-values were calculated using t-tests for continuous variables and chi-square tests for categorical variables; *p*-values are presented for descriptive purposes and were not adjusted for multiple comparisons. Physical activity represents total minutes per week; MET, metabolic equivalent of task (total minutes per week). Depression categories based on PHQ-9 scores: none (0-4), mild (5-9), moderate (10-14), severe (≥15). Family PIR categories: low (<1.3), middle (≥1.3 to <3.5), high (≥3.5). BMI, body mass index; PIR, poverty-to-income ratio; DII, dietary inflammatory index; PUFA, polyunsaturated fatty acids; HDL-C, high-density lipoprotein cholesterol; MET, metabolic equivalent of task.

### 3.2 Logistic regression analysis

Table 2 presents the associations between depression, DII, PIR, and constipation across three regression models. In the fully adjusted model (Model 3), higher depression scores were independently associated with increased constipation risk (OR: 1.04, 95% CI: 1.03–1.06, *p* < 0.0001). Compared to individuals without depression, those with mild, moderate, and severe depression exhibited progressively elevated odds of constipation, with odds ratios of 1.49 (95% CI: 1.23–1.82), 1.81 (95% CI: 1.37–2.40), and 1.72 (95% CI: 1.22–2.44), respectively (p for trend <0.0001). Similarly, DII levels were positively correlated with constipation risk. Each unit increase in DII was associated with a 15% higher odds of constipation (OR: 1.15, 95% CI: 1.04–1.27, *p* = 0.0064). Participants in the second, third, and highest DII quartiles showed significantly greater risk with odds ratios of 1.45 (95% CI: 1.07–1.95), 1.47 (95% CI: 1.03–2.11), and 1.73 (95% CI: 1.13–2.64), respectively, compared to the lowest quartile (p for trend = 0.035). For PIR, individuals in the highest group (≥3.5) had a significantly lower constipation risk (OR: 0.77, 95% CI: 0.62–0.97, *p* = 0.0292) than those in the lowest group, with a significant inverse trend (p for trend = 0.0329). These associations remained robust after extensive adjustment for demographic factors, socioeconomic status, comorbidities, lifestyle variables, and nutritional parameters.

**Table 2:** Association between depression, DII, PIR, and constipation.

Characteristics	Model 1		Model 2		Model 3	
	OR (95% CI)	*p*-value	OR (95% CI)	*p*-value	OR (95% CI)	*p*-value
Depression score						
Continues	1.06 (1.05, 1.07)	<0.0001	1.05 (1.03, 1.06)	<0.0001	1.04 (1.03, 1.06)	<0.0001
Severity of depression						
No depression	Ref		Ref		Ref	
Mild depression	1.51 (1.30, 1.75)	<0.0001	1.36 (1.17, 1.58)	<0.0001	1.49 (1.23, 1.82)	<0.0001
Moderate depression	2.19 (1.78, 2.69)	<0.0001	1.92 (1.56, 2.37)	<0.0001	1.81 (1.37, 2.40)	<0.0001
Severe depression	2.25 (1.73, 2.92)	<0.0001	1.87 (1.43, 2.44)	<0.0001	1.72 (1.22, 2.44)	0.0022
P for trend	<0.0001		<0.0001		<0.0001	
DII						
Continues	1.24 (1.20, 1.29)	<0.0001	1.17 (1.13, 1.21)	<0.0001	1.15 (1.04, 1.27)	0.0064
Q1	Ref		Ref		Ref	
Q2	1.70 (1.41, 2.05)	<0.0001	1.56 (1.29, 1.88)	<0.0001	1.45 (1.07, 1.95)	0.0156
Q3	2.11 (1.76, 2.53)	<0.0001	1.73 (1.44, 2.08)	<0.0001	1.47 (1.03, 2.11)	0.0353
Q4	2.74 (2.29, 3.27)	<0.0001	2.10 (1.75, 2.52)	<0.0001	1.73 (1.13, 2.64)	0.011
P for trend	<0.0001		<0.0001		0.0350	
PIR						
Continues	0.86 (0.83, 0.89)	<0.0001	0.87 (0.84, 0.91)	<0.0001	0.95 (0.90, 1.01)	0.0852
<1.3	Ref		Ref		Ref	
≥1.3 and <3.5	0.77 (0.67, 0.88)	0.0002	0.79 (0.69, 0.91)	0.0012	0.93 (0.77, 1.11)	0.4155
≥3.5	0.53 (0.45, 0.62)	<0.0001	0.56 (0.48, 0.66)	<0.0001	0.77 (0.62, 0.97)	0.0292
P for trend	<0.0001		<0.0001		0.0329	

Model 1: unadjusted; Model 2: adjusted for age, sex, and ethnicity; Model 3: additionally adjusted for marital status, PIR, depression score, education level, smoking status, alcohol consumption, BMI, hypertension, diabetes, hyperlipidemia, DII, total cholesterol, HDL-C, fiber, PUFA, vitamin D, and Mg; DII dietary inflammatory index; PIR, poverty-to-income ratio; OR, odds ratio; CI, confidence interval.

### 3.3 Dose-response relationship between depression and constipation risk

**Figure 2:**
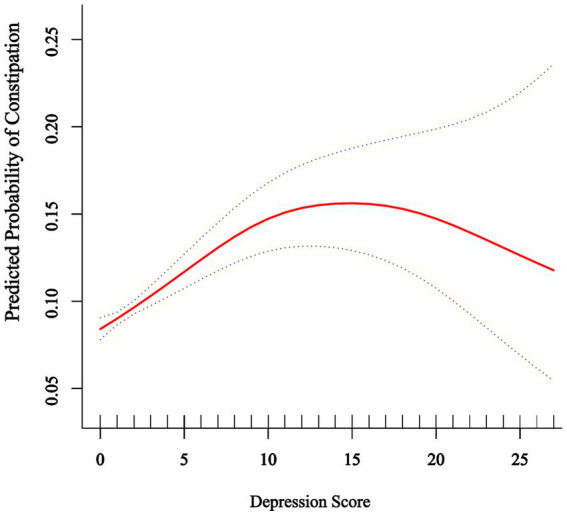
Association between depression score and predicted probability of constipation. The solid red line represents the predicted probability of constipation across the range of depression scores (0–27) based on a restricted cubic spline model with four knots. The dotted lines represent the 95% confidence intervals. The model was adjusted for age, sex, ethnicity, marital status, poverty income ratio, education level, smoking status, alcohol consumption, BMI, hypertension, diabetes mellitus, hyperlipidemia, DII, total cholesterol, HDL-C, fiber, PUFA, vitamin D, and Mg.

Restricted cubic spline (RCS) regression identified a non-linear dose-response relationship between depression and constipation risk (Figure 2), featuring an inflection point at a depression score of 10 (P for non-linearity = 0.001). For PHQ-9 scores below 10, increasing depression severity was significantly associated with higher constipation risk (OR: 1.08, 95% CI: 1.05–1.11, *p* < 0.0001), indicating an 8% increase in risk per one-point rise in PHQ-9 score. Importantly, the risk plateaued at scores above 10, with no significant additional risk observed with further increases in depression severity (OR: 0.98, 95% CI: 0.93–1.02, *p* = 0.3018). The significant slope difference (OR: 0.90, 95% CI: 0.85–0.96, *p* = 0.0014) confirmed the threshold model’s superior fit over linear alternatives, with the likelihood ratio test demonstrating significant improvement compared to the linear model (*p* = 0.001) (Table 3).

**Table 3:** Threshold effect analysis of depression score on constipation risk.

Variable	OR (95% CI)	*p*-value
Model I		
Linear effect of depression score	1.04 (1.03, 1.06)	<0.0001
Model II		
Depression score <10	1.08 (1.05, 1.11)	<0.0001
Depression score <10	0.98 (0.93, 1.02)	0.3018
Slope difference (≥10 vs. < 10)	0.90 (0.85, 0.96)	0.0014

The breakpoint (K) was 10 in Model II; the predicted value of the equation at the breakpoint was-1.58 (95% CI: −1.70, −1.34); the *p*-value for the log likelihood ratio test comparing Model I and Model II was 0.001. All models were adjusted for age, sex, ethnicity, marital status, poverty income ratio, education level, smoking status, alcohol consumption, BMI, hypertension, diabetes mellitus, hyperlipidemia, DII, total cholesterol, HDL-C, fiber, PUFA, vitamin D, and Mg.

### 3.4 Statistical mediation analysis

**Figure 3:**
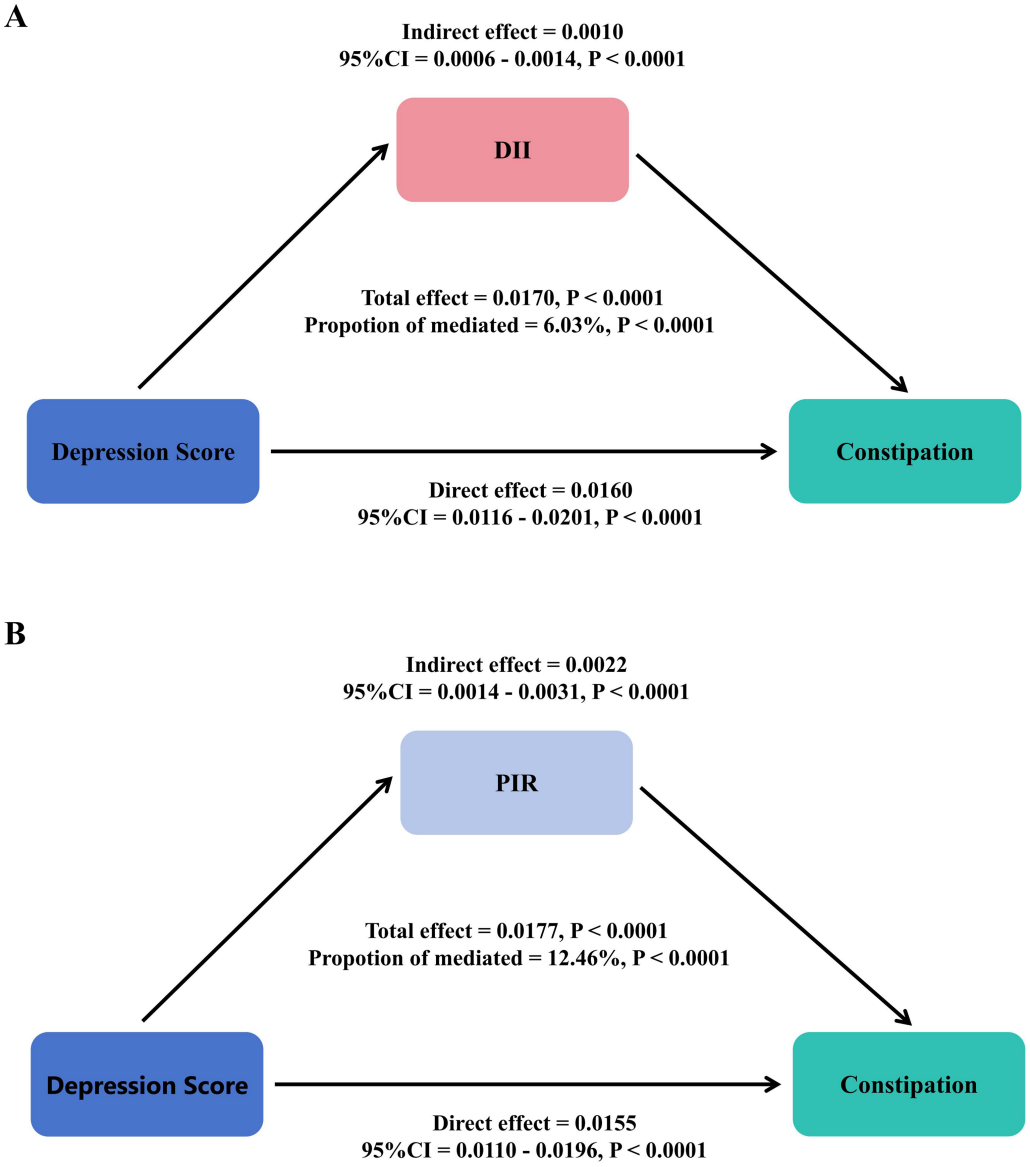
Statistical mediation analysis examining the relationship between depression, DII **(A)**, PIR **(B)**, and constipation.

Statistical mediation analysis revealed that DII statistically mediated 6.03% and PIR statistically mediated 12.46% of the observed association between depression and constipation after covariate adjustment (Figure 3).

### 3.5 Subgroup analysis

**Figure 4:**
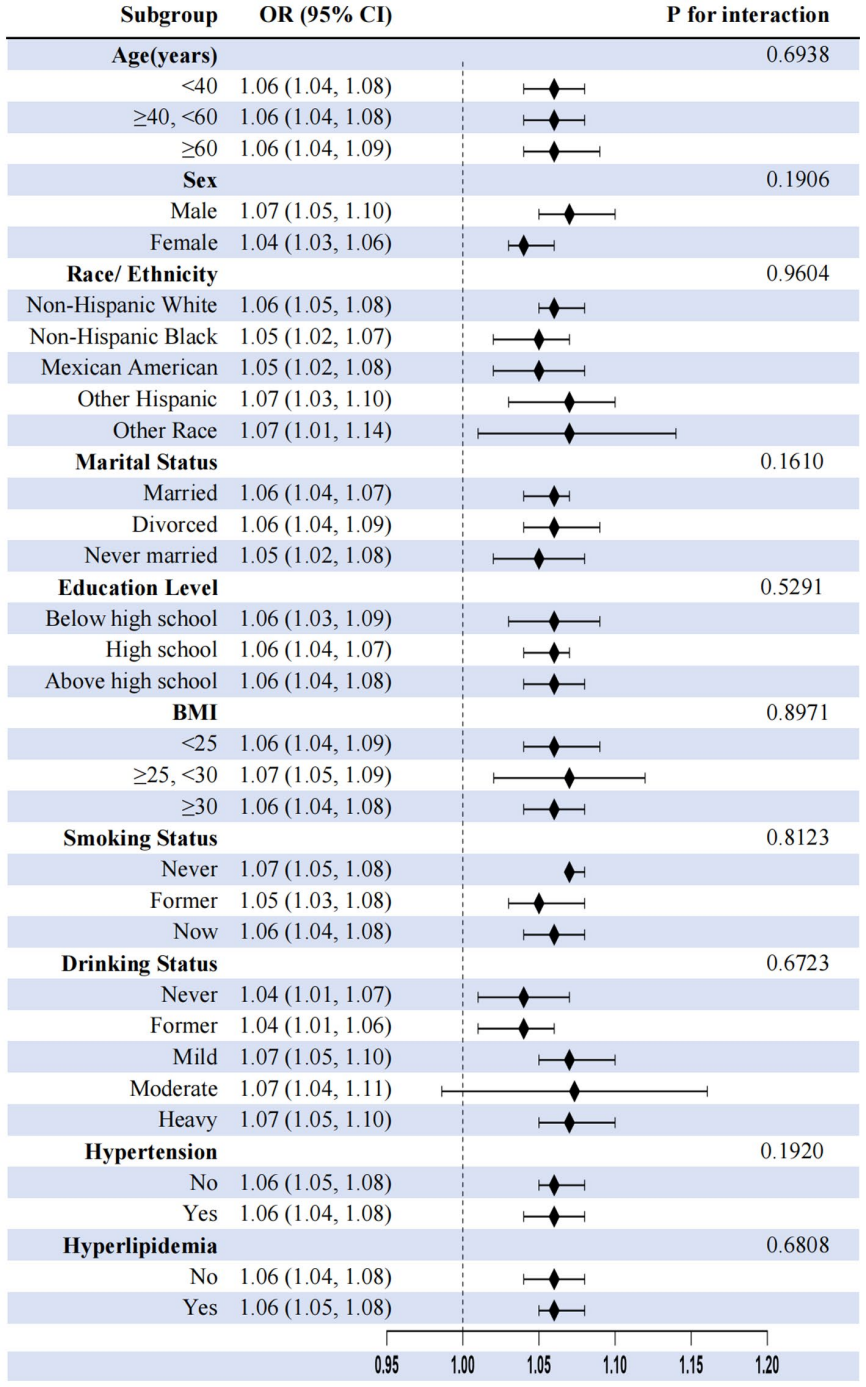
Subgroup analysis of associations between depression and constipation.

Subgroup analyses consistently demonstrated depression–constipation associations across all demographic and clinical subgroups (OR range: 1.04–1.14, all *p* < 0.05), with no significant effect modification by covariates, including age, sex, ethnicity, marital status, education level, BMI, smoking status, drinking status, hypertension, or hyperlipidemia (all interaction *p* > 0.05), highlighting robust universal applicability (Figure 4).

## 4 Discussion

This cross-sectional study revealed a non-linear threshold association between depression and constipation risk using restricted cubic spline models. Each 1-unit increase in PHQ-9 score was associated with a 4% increase in constipation risk (OR = 1.04, 95% CI: 1.03–1.06). Specifically, for PHQ-9 scores below 10, each unit increase conferred an 8% risk elevation (OR = 1.08, 95% CI: 1.05–1.11), while scores above this threshold showed no additional significant risk (OR = 0.98, 95% CI: 0.93–1.02, *p* = 0.3018), indicating a plateau effect. Individuals with severe depression demonstrated significantly elevated constipation odds (OR = 1.72, 95% CI: 1.22–2.44). Meanwhile, pro-inflammatory diets (highest DII quartile: OR = 1.73, 95% CI: 1.13–2.64) were positively associated with constipation, while higher socioeconomic status (highest PIR category: OR = 0.77, 95% CI: 0.62–0.97) was inversely associated. Mediation analysis further indicated that DII and PIR statistically explained 6.03 and 12.46%, respectively, of the association between depression and constipation.

Our findings of a significant positive association between depression and constipation (OR: 1.04 per PHQ-9 point) align with previous NHANES studies (OR = 2.20, 95% CI: 1.68–2.87) (46). The novel contribution of our analysis is identifying the non-linear threshold effect at PHQ-9 = 10, corresponding to moderate depression severity. This threshold likely reflects converging biological mechanisms: from a neuroendocrine perspective, it may indicate the maximum impact of cortisol’s inhibitory effects on gastrointestinal motility (47, 48); from an inflammatory standpoint, moderate depression (PHQ-9 ≥ 10) is associated with significantly elevated inflammatory markers including CRP, IL-6, and TNF-α (49, 50); and from a neurophysiological perspective, PHQ-9 = 10 often marks pronounced autonomic nervous system dysfunction affecting vagal tone and gut motility (51). Additionally, individuals with moderate depression often experience substantial changes in physical activity, medication use, and dietary habits that collectively contribute to constipation without proportional increases at higher severity levels (52).

The depression–constipation relationship appears to operate through multiple interconnected pathways, with our mediation analysis revealing partial roles for both dietary inflammation (DII: 6.03%) and socioeconomic factors (PIR: 12.46%). These “modest” mediation proportions highlight the multifactorial nature of this relationship. Mechanistically, depression and constipation are linked through the central nervous system–gut axis: depression modulates vagal tone and serotonin balance, affecting intestinal motility (53), while dysregulated corticotropin-releasing factor and disrupted gut microbiota alter short-chain fatty acid production and gut health (54). Inflammatory pathways further connect these conditions, with DII influencing both depression (through inflammatory markers affecting neurotransmitter metabolism and neuroplasticity) (55, 56) and constipation (by reducing beneficial gut bacteria, increasing intestinal permeability, and inhibiting motility) (57, 58). This creates a bidirectional cycle where gut microbiota alterations affect neurotransmitters and inflammatory mediators, exacerbating depressive symptoms, while depression further aggravates constipation through altered intestinal autonomic function (67, 68). Socioeconomic factors compound these effects, with low-income individuals facing nutritional inequity and limited access to anti-inflammatory foods (59, 60) while experiencing chronic stress-induced glucocorticoid resistance and heightened inflammatory responses (61, 62). Our observed DII mediation proportion (6.03%) is lower than the 71.43% reported for specific saturated fatty acids in depression models (63), likely reflecting methodological differences between controlled nutrient studies versus our population-based approach capturing holistic dietary patterns.

This study has several methodological strengths that enhance result reliability. We utilized a large nationally representative sample (NHANES 2005–2010; *n* = 12,854), employed restricted cubic spline modeling to identify non-linear relationships, and used mediation analysis to examine the potential mediating relationships involving DII and PIR, elucidating the relative contributions of dual mediation mechanisms within a single study for the first time. Furthermore, we implemented progressive multivariable adjustment strategies controlling for an extensive array of potential confounders across demographic (age, sex, and ethnicity), socioeconomic (education, marital status, and PIR), lifestyle (smoking, alcohol consumption, and physical activity), clinical (BMI, diabetes, hypertension, and hyperlipidemia), and nutritional (total cholesterol, HDL-C, fiber, polyunsaturated fatty acids, vitamin D, and magnesium) factors and performed comprehensive subgroup analyses verifying result robustness across different populations.

While this study makes significant contributions, several limitations should be acknowledged: (1) the cross-sectional design precludes establishing causality between depression and constipation; (2) results based on U.S. adults may not generalize to populations from other regions; (3) constipation and dietary data obtained from questionnaires could be affected by recall bias, with our operational definitions of frequency terms representing reasonable but arbitrary thresholds that influence case identification; (4) despite adjusting for numerous confounders, unmeasured factors (particularly antidepressant use and comorbid irritable bowel syndrome) may influence the observed associations; (5) depression assessment using PHQ-9 at a single time point may not capture symptom fluctuations; and (6) the absence of objective inflammatory biomarkers (such as C-reactive protein, interleukin-6, or tumor necrosis factor-*α*) limits our ability to validate the biological relevance of DII scores and may introduce measurement error in assessing dietary inflammatory potential.

In conclusion, this study identified a non-linear threshold association between depression and constipation, with a significant inflection point at a PHQ-9 score of 10. Below this threshold, each unit increase in depression score conferred an 8% elevation in constipation risk, while scores above 10 demonstrated a plateau effect with no additional significant risk increase. Dietary inflammation and socioeconomic factors partially mediated this relationship, suggesting potential intervention targets. These findings emphasize the importance of constipation risk screening in patients with depression, particularly those with moderate depression symptoms (PHQ-9 ≥ 10), as this represents the threshold where maximal risk is achieved. As our findings are derived from a cross-sectional design, we cannot establish temporal ordering or causality between depression and constipation. Future research should explore causal relationships through longitudinal designs; implement methodological approaches to address unmeasured confounding, such as collecting comprehensive medication data and conducting sensitivity analyses; and incorporate objective inflammatory biomarkers and gastrointestinal function measures to reduce measurement bias and validate self-reported assessments.

## 5 Conclusion

Higher depression severity was linked to greater constipation risk (particularly for PHQ-9 ≥ 10), partially mediated by DII and PIR.

## Data Availability

Publicly available datasets were analyzed in this study. This data can be found at: https://wwwn.cdc.gov/nchs/nhanes/default.aspx.
